# Enhanced cytokinin signaling stimulates cell proliferation in cambium of Populus

**DOI:** 10.1186/1753-6561-5-S7-O23

**Published:** 2011-09-13

**Authors:** Juha Immanen, Kaisa Nieminen, Jing Zhang, Rishi Bhalerao, Ykä Helariutta

**Affiliations:** 1 University of Helsinki, Finland; 2 University of Lausanne, Switzerland; 3 Umeå Plant Science Center, Sweden

## Introduction

Understanding the regulation of radial growth that underlies wood development is of great importance for future use of tree products as a renewable resource. For this purpose, we have performed a detailed analysis of cytokinin function in the regulation of cambial development in a tree stem. Furthermore, we have identified potential cytokinin signaling response genes active during the secondary development in Arabidopsis and currently studying the best candidate genes also in Populus.

## Results and discussion

 In the cambium, expression of cytokinin receptor genes peaks in the actively dividing cells. This indicates that cytokinin signaling pathway is a major regulator of cambial cell proliferation. We have previously shown in transgenic Populus trees that we can slow down the cambial activity by reducing the level of cytokinin signaling in it [1]. To study if cytokinin signaling is truly a rate-limiting process for cambial activity, we have now engineered transgenic Populus trees with an enhanced cambial cytokinin signaling level. In these trees, we were able to see that elevated cytokinin signaling stimulated the cambial cell division activity and led to increased biomass production. Furthermore, based on microarray profiling in Arabidopsis, we have been able to identify several transcription factors functioning downstream of cytokinin signaling during the secondary development. These transcriptional regulators have stimulatory effect on radial growth in Arabidopsis root. We are currently studying if the Populus orthologs of these genes have similar function in the cambium of the tree stem.

## Conclusions

We have shown that the cambial cell division activity, and through it the production of stem biomass, can be enhanced or decreased through elevated or decreased cytokinin signaling. Together, our results show that cytokinins are major rate-limiting hormonal regulators of cambial development. Our study provides potential for applied research towards future breeding of faster growing tree cultivars.
